# Mapping nanoscale carrier confinement in polycrystalline graphene by terahertz spectroscopy

**DOI:** 10.1038/s41598-024-51548-z

**Published:** 2024-02-07

**Authors:** Patrick R. Whelan, Domenico De Fazio, Iwona Pasternak, Joachim D. Thomsen, Steffen Zelzer, Martin O. Mikkelsen, Timothy J. Booth, Lars Diekhöner, Ugo Sassi, Duncan Johnstone, Paul A. Midgley, Wlodek Strupinski, Peter U. Jepsen, Andrea C. Ferrari, Peter Bøggild

**Affiliations:** 1 DTU Physics, Technical University of Denmark, Fysikvej, Bld. 309, 2800 Kongens Lyngby, Denmark; 2 Department of Materials and Production, Aalborg University, Skjernvej 4A, 9220 Aalborg, Denmark; 3 Cambridge Graphene Centre, University of Cambridge, 9 JJ Thomson Avenue, Cambridge, CB3 0FA UK; 4 Department of Molecular Sciences and Nanosystems, Ca’ Foscari University of Venice, 30172 Venice, Italy; 5 Faculty of Physics, Warsaw University of Technology, Koszykowa 75, 00-662 Warsaw, Poland; 6 Vigo System S.A., 129/133 Poznanska Str, 05-850 Ozarow Mazowiecki, Poland; 7 Center for Nanostructured Graphene (CNG), Technical University of Denmark, Ørsteds Plads 345C, 2800 Kongens Lyngby, Denmark; 8 Department of Materials Science and Metallurgy, University of Cambridge, 27 Charles Babbage Road, Cambridge, CB3 0FS UK; 9 DTU Fotonik, Technical University of Denmark, Ørsteds Plads 343, 2800 Kongens Lyngby, Denmark

**Keywords:** Electronic properties and devices, Two-dimensional materials

## Abstract

Terahertz time-domain spectroscopy (THz-TDS) can be used to map spatial variations in electrical properties such as sheet conductivity, carrier density, and carrier mobility in graphene. Here, we consider wafer-scale graphene grown on germanium by chemical vapor deposition with non-uniformities and small domains due to reconstructions of the substrate during growth. The THz conductivity spectrum matches the predictions of the phenomenological Drude–Smith model for conductors with non-isotropic scattering caused by backscattering from boundaries and line defects. We compare the charge carrier mean free path determined by THz-TDS with the average defect distance assessed by Raman spectroscopy, and the grain boundary dimensions as determined by transmission electron microscopy. The results indicate that even small angle orientation variations below 5° within graphene grains influence the scattering behavior, consistent with significant backscattering contributions from grain boundaries.

## Introduction

Production of single layer graphene (SLG) on a large scale by chemical vapor deposition (CVD) is reaching industrial maturity^1–7^. In most cases, this involves a subsequent transfer process, where SLG is moved to a target substrate, such as oxidized Si or polymer^6–11^. As this process can result in contamination of and damage to SLG^12^, significant efforts have been made to identify routes to circumvent this step^13–16^. CVD most often leads to polycrystalline growth, where the presence of grain boundaries (GBs) impair transport properties^3–5^. Wafer-scale graphene growth has been pursued by enabling multiple fully or nearly co-oriented grains to grow together to form single-crystals^4,5,17^. It is relevant to investigate how sensitive electron transport is to the grain size distribution, as well as to orientation angle distribution.

Industrial applications of SLG require a systematic approach to large-area characterization of properties such as coverage, defect density, and electrical characteristics. This is also crucial for development of new growth recipes, for monitoring of post-process influence on device key performance indicators, for quality control during production, and for studies of transport mechanisms on a more fundamental level.

Practical and industrially relevant characterization methods for spatially mapping the properties of wafer-scale SLG are emerging. These include automated quantitative optical microscopy^18^, Raman spectroscopy^19–22^, fast-turn-around device fabrication and characterization schemes^23^, scanning micro four-point probes^24–26^, as well as far-field mapping techniques based on terahertz time-domain spectroscopy (THz-TDS)^27–32^, eddy currents, and microwave impedance measurements^32^, Using such methods it is possible to obtain spatial information about coverage, defect density, number of layers, conductivity, doping, strain, and carrier mobility, all of which are essential for the optimization of growth, transfer and device fabrication.

Raman spectroscopy is an integral part of graphene research. It is used to determine the number and orientation of layers^21^, the quality and types of edges^33^, and the effects of perturbations^20^, such as electric^34^ and magnetic fields^35,36^, strain^22^, doping^34,37,38^, disorder^39,40^ and functional groups^41^. In terms of electrical properties, THz-TDS gives reliable estimates of not just conductivity, but also carrier density and mobility^42–44^, as well as electrical continuity of SLG^25,32,45^, while being able to rapidly scan wafer-sized samples, i.e. 300 mm graphene wafer with a 1 mm step size in 1 h^32,45^. Far-field THz-TDS is thus excellently suited for large-scale, high-speed mapping of the electrical properties of graphene films. However, due to the ~ 0.1–1 mm size diffraction limited spotsize^32^, it is not able to provide useful spatial information on the sub-mm scale. This analysis, however, relies on the Drude-like form of the THz optical conductivity^46^, arising from the predominance of intra-band scattering in this frequency range^46^. Fitting the frequency-dependent Drude conductivity to the THz conductivity allows one to extract the scattering time (momentum relaxation time) and the DC (low-frequency) conductivity^32^. Using the semiclassical Boltzmann equation under the assumption of diffusive transport^47^, the carrier density and scattering time can then be estimated^42^. For uniformly conducting SLG, the electrical properties derived from THz-TDS using the Drude model generally agree well with conventional contact-based electrical measurements.^26,30,31,42,44,45^.

The Drude model, however, assumes isotropic scattering^48^, where by every scattering event randomizes the carrier momentum. This model is adequate when the distance between fully or partially reflecting line defects (such as grain boundaries (GBs)) is significantly larger than the transport lengths, i.e. the carrier mean free path and the characteristic THz electron diffusion length that are both ~ 20–100 nm for typical CVD SLG on SiO_2_ and THz spectroscopic frequency ranges^32,45^.

Polycrystalline SLG with small grains below a few µm may change this picture. Here, a high of GBs density may act as reflecting or partially reflecting line defects, altering overall scattering behavior and momentum distribution^49–52^. In this case, the conductivity spectrum is better described by the phenomenological Drude–Smith (DS) model^25,53^. This introduces a backscattering parameter, -1 ≤ *c* ≤ 0, which represents the overall degree of preferential backscattering experienced by charge carriers thus, indirectly, the effective density of GBs and other line defects^53^.

Here we spatially map *c* in SLG and extract the carrier mean free path from THz-TDS measurements. We show that this is in good agreement with the defect density derived from Raman spectroscopy. The mean free path is further compared to the SLG grain size from transmission electron microscopy (TEM). Our results suggest that even small angle misalignments < 5° between SLG grains may have a large impact on the carrier scattering behavior.

## Experimental

SLG is grown by CVD on 3 µm thin film Ge on Si^54,55^. Surface reconstruction of the growth substrate leads to formation of a highly faceted Ge surface pattern after the CVD process^55^. For this reason, this type of SLG is polycrystalline with a small grain size, allowing us to correlate information on the submicron grain structure extracted from THz-TDS with three local characterisation techniques: scanning tunnelling microscopy (STM), Raman spectroscopy and selective electron diffraction (SED). STM analysis indicates that the electron local density of states of these SLG films can vary down to a sub-100 nm length scale^55^.

SLG is then transferred from Ge(100)/Si(100) onto 4-inch high-resistivity Si (HR-Si, *ρ* > 10 kΩ·cm) by electrochemical delamination in aqueous solution (2 M potassium chloride) with samples submerged at a rate of 1 mm/s and − 10 to − 30 V applied relative to a carbon counter electrode^56^.

THz-TDS measurements are performed using a commercial fiber-coupled Picometrix spectrometer^30^. The sample is raster-scanned in the focal plane of the THz beam at normal incidence to form a spatial map with a 200 μm step size and a diffraction-limited spot size ~ 350 μm at 1 THz. The complex frequency-dependent SLG sheet conductivity, $\tilde{\sigma }_{{\text{s}}} (\omega ) = \sigma_{1} + i\sigma_{2}$, can be determined for each pixel in the scanned map from the transmission function $\tilde{T}_{{{\text{film}}}} (\omega ) = \tilde{E}_{{{\text{film}}}} (\omega )/\tilde{E}_{E} (\omega )$, where $\tilde{E}_{{{\text{film}}}} (\omega )$ and $\tilde{E}_{{{\text{sub}}}} (\omega )$ are the Fourier transforms of the THz waveforms transmitted through SLG-covered and non-covered Si, respectively^32^. $\tilde{\sigma }_{{\text{s}}} (\omega )$ is extracted from the transmission function after one internal reflection inside the Si substrate as:^25^

1 $$\tilde{\sigma }_{{\text{s}}}^{{(1{\text{st}})}} (\omega ) = \frac{{n_{{\text{A}}} \sqrt {n_{{\text{A}}}^{2} + 4n_{{\text{A}}} n_{{\text{B}}} \tilde{T}_{{{\text{film}}}}^{{(1{\text{st}})}} (\omega ) + 4n_{{\text{B}}}^{2} \tilde{T}_{{{\text{film}}}}^{(1E)} (\omega )} - n_{{\text{A}}}^{2} - 2n_{{\text{A}}} n_{{\text{B}}} \tilde{T}_{{{\text{film}}}}^{{(1{\text{st}})}} (\omega )}}{{2n_{{\text{B}}} Z_{0} \tilde{T}_{{{\text{film}}}}^{{(1{\text{st}})}} (\omega )}},$$

where *Z*_0_ is the vacuum impedance, *n*_A_ = $\tilde{n}_{{{\text{sub}}}} + 1$ and *n*_B_ = $\tilde{n}_{{{\text{sub}}}} - 1$ for a substrate with refractive index $\tilde{n}_{{{\text{sub}}}}$. Timing jitter corrections are used to increase the accuracy of the electrical properties determined from THz-TDS^57^. Two 4-inch wafers are prepared and measured by THz-TDS.

Raman spectra are measured with a Thermo Fisher DXRxi spectrometer using a 532 nm laser at 1 mW power, acquiring 20 scans per point. The spot size is ~ 1 µm.

The distribution of SLG grain size, ℓ_C_, is mapped by SED^58^, after transferring SLG onto TEM grids^59,60^. SED is performed in a scanning TEM and involves the acquisition of an electron diffraction pattern at each probe position in a two-dimensional scan region^61^, using a Philips CM300 field emission gun TEM operated at 50 kV, well below the threshold for SLG knock-on damage^62^, and fitted with a NanoMegas Digistar system. This enables the simultaneous scan and acquisition of diffraction patterns with an external optical charge coupled device imaging the phosphor viewing screen of the microscope. In this way, nanobeam electron diffraction patterns are acquired with a step size ∼10 nm.

The resulting SED dataset is analysed to determine the grain structure. First, a spatially averaged diffraction pattern for the scanned region is calculated by summing all patterns, in analogy to selected area electron diffraction^63,64^. Diffraction contrast images^63^ are formed from the SED dataset by plotting the intensity within a selected subset of pixels in each diffraction pattern as a function of probe position^61,65^. Quantitative mapping of the crystallographic orientation at each probe position is done by template matching^66^, whereby each diffraction pattern is compared to a library of patterns simulated for all possible orientations to find the best match. The resulting orientation maps are analysed with the MTEX MATLAB toolbox^67^. A median filter is applied to remove spurious single pixels arising due to mis-indexation and GBs are then defined where the pixel-to-pixel angular deviation is > 5°. GBs are extracted and the distance between boundary points is determined using the “test line intercept method”, in which a series of random straight lines are drawn across the GB structure and the distance between the intersections of the boundary lines with the test lines is measured^68^.

STM is performed on SLG transferred to n-doped (*ρ* < 0.025 Ω·cm) Si(100)^59,60^. Samples are annealed for 5 h at 500 °C at ultra-high vacuum to remove polymer residues after transfer. STM is done at ~ 80 K and with − 500 mV sample bias applied with a mechanically cut Pt-Ir tip.

We note that, while STM and SED extract spatial information by direct imaging of SLG, both Raman spectroscopy and THz-TDS relly on analysis methods to obtain the weighted average of transport lengths and grain structure across the diffraction limited spot sizes, which are ~ 0.5 μm for Raman spectroscopy and 350 mm for THz-TDS.

## Results and discussion

**Figure 1:**
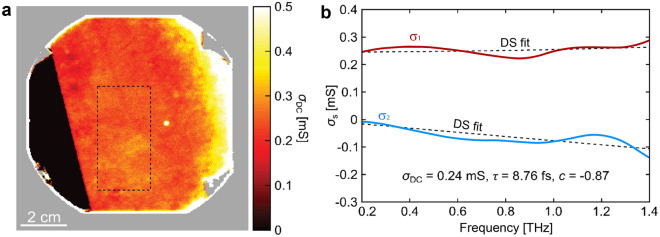
THz-TDS measurement of SLG transferred from Ge to HR-Si. (**a**) Map of *σ*_DC_ for SLG on a 4-inch Si wafer. (**b**) $\tilde{\sigma }_{{\text{s}}} (\omega )$ spectra from a pixel in (**a**) with fits to the DS model.

CVD SLG grown on thin film Ge and transferred to 4-inch HR-Si is measured with THz-TDS. *σ*_DC_ appears uniform in the central region of the wafer, Fig. 1a. A plot of $\tilde{\sigma }_{{\text{s}}} (\omega )$ from a single measurement point on the map (Fig. 1b) shows the real and imaginary parts of the conductivity to be increasing and decreasing, respectively. This DS behavior is characteristic of discontinuous SLG, i.e. with a significant amount of fully or partially reflecting line defects. In the DS model, $\tilde{\sigma }_{{\text{s}}} (\omega )$ can be written as:^25,53,69^

2 $$\tilde{\sigma }_{{\text{s}}} \left( \omega \right) = \frac{{W_{{\text{D}}} }}{1 - i\omega \tau }\left( {1 + \frac{c}{1 - i\omega \tau }} \right),$$

where *τ* is the scatttering time*, W*_D_ is the Drude weight, related to the DC conductivity as *σ*_DC_ = *W*_D_(1 + *c*), and *c* is the backscattering parameter, −1 ≤ *c* ≤ 0^25,53,69^. If *c* = 0, the Drude model is recovered, while *c* =  − 1 corresponds to maximum backscattering, where *σ*_DC_ = 0^69^. The carrier mean free path, ℓ_mfp_, can be determined from *τ* as ℓ_mfp_ = *ν*_F_*τ*^25^, where *ν*_F_ is the Fermi velocity. Using a substrate (Si) with dielectric constant *ε*_s_ = 11.67^70^ and for a doping level  ~ 1·10^13^ × cm^−2^, as estimated from Raman spectroscopy, *ν*_F_ is ~ 1.0 × 10^6^ m/s^44^.

The DS model is fitted to $\tilde{\sigma }_{{\text{s}}} (\omega )$ for all measurement points inside the region highlighted in Fig. 1a (∼7200 pixels). For the representative example in Fig. 1b, the DS model is fitted to the measured $\tilde{\sigma }_{{\text{s}}} (\omega )$ with *c* =  − 0.88, *τ* = 8.76 fs and *σ*_DC_ = 0.24 mS, which shows that carriers in the corresponding region are experiencing preferential backscattering.

**Figure 2:**
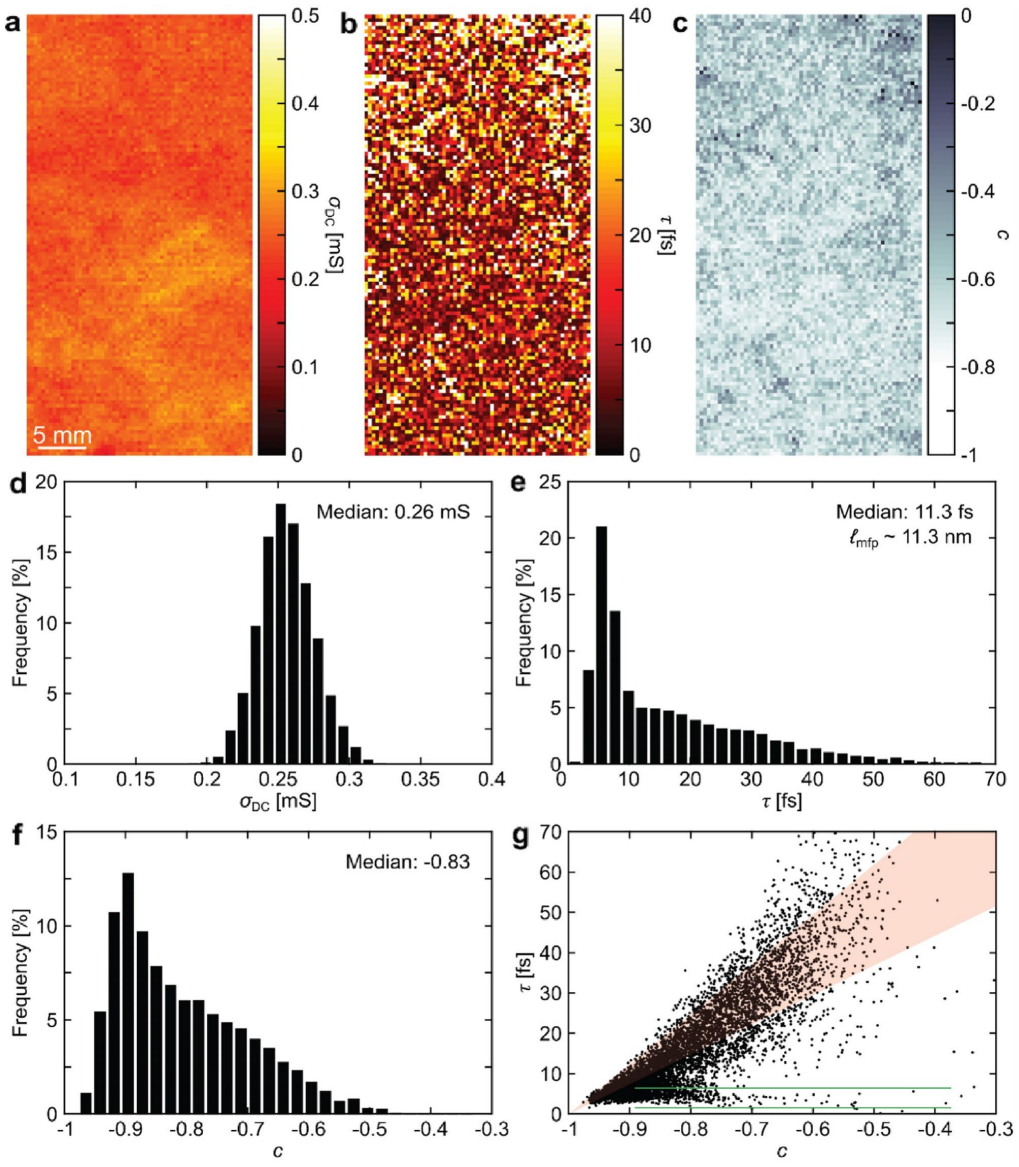
Maps and histograms of (**a**, **d**) *σ*_s,DC_, (**b**, **e**) *τ* and (**c**, **f**) *c*. (**g**) Correlation plot of *c* and *τ*, showing a dominant band (red region) and a weaker horizontal band (between green lines). All data are extracted from the pixels in the highlighted rectangle in Fig. 1a.

Figures 2a–f show maps and histograms of *σ*_DC_, *τ* and *c* from the highlighted area in Fig. 1a. From the median *τ* we determine ℓ_mfp_ = *ν*_F_*τ* =  ~ 11.3 nm. The median is -0.83 for *c*, not far from the case of fully reflective boundaries or defects (*c* =  − 1). The microscopic origins of the DS and modified DS models are described in Ref.^71^. These models, as well as others^72–74^, could in principle be extended to polycrystalline SLG and provide more detailed and quantitative information on the relation between scattering processes and THz response, but this has not yet been done, to the best of our knowledge.

The maps in Fig. 2b,c indicate that *c* moves towards 0 where *τ* is highest. In the correlation plot of *c* and *τ* in Fig. 2g, *τ* decreases towards 0 as charge carriers are increasingly backscattered, i.e., as *c* approaches − 1. This supports the notion that the preferential backscattering originates from GBs, lowering the carrier mean free path^75,76^. Without GBs or extended damage, the momentum relaxation time and carrier mobility of CVD SLG is typically limited by impurity scattering, often originating from traps, contamination and water, all of which maintain a Drude-like behavior^25^. The distribution is fan-shaped, with a tendency of splitting into a dominant band (Fig. 2g, red region), as well as a fainter scatter of points with a very short scattering time ~ 3–4 fs, independent on c (Fig. 2g. between green lines). The substructure in the distribution could be related to slight non-uniformities in the region of interest (sample zone in Fig. 1a. marked with a dashed rectangle). The large spread in extracted scattering times (Fig. 2e) corresponds to a variation of mean free path, as v_F_ is constant in SLG, ~$10^{6} \,{\text{m/s}}$, so that 10 fs translates to 10 nm. The distribution of mean free paths in Fig. 2g thus extends to 60 nm.

**Figure 3:**
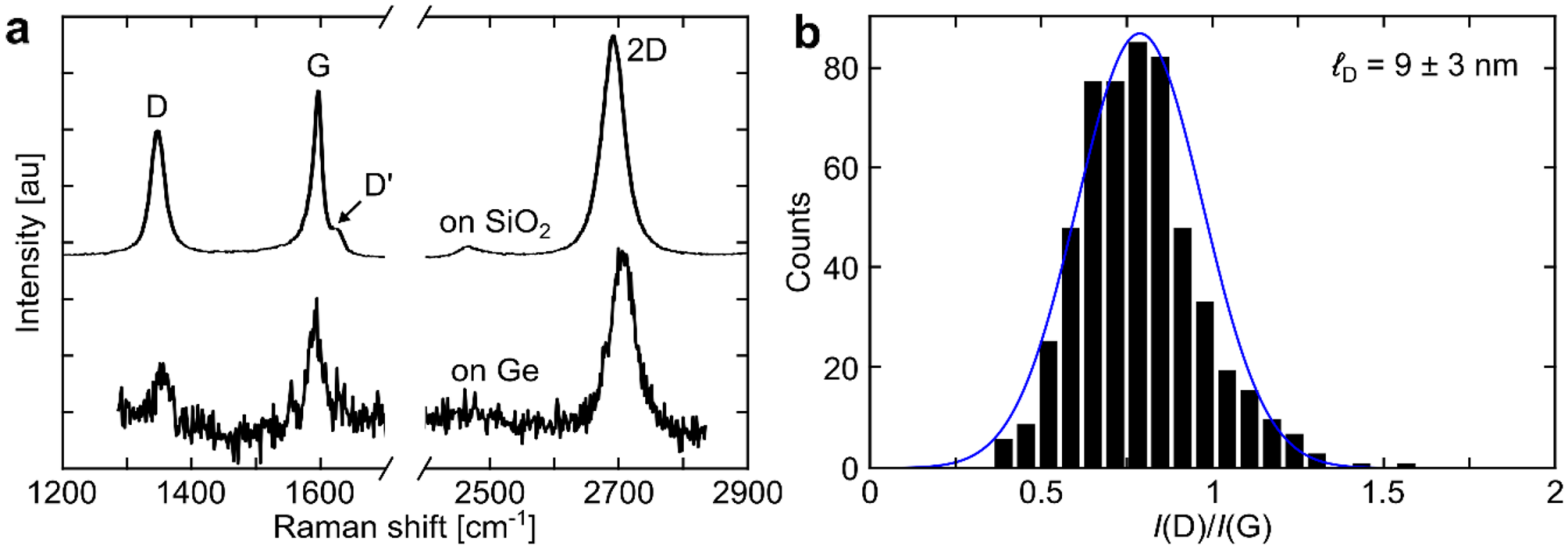
(**a**) Representative Raman spectra of as-grown SLG on Ge and after transfer to Si/SiO_2_. (**b**) Histogram of *I*(D)/*I*(G). The blue curve is a fitted normal distribution.

Raman characterization of Ge-grown SLG transferred to Si/SiO_2_ reveals a substantial D-peak (Fig. 3a), indicating a high defect density^19^. A Raman spectrum of as-grown SLG on Ge is shown for comparison, where the D-peak is already present. From the Raman D to G peak intensity ratio (Fig. 3b), *I*(D)/*I*(G), the average distance between defects, ℓ_D_, can be determined as ℓ_D_^2^ (in nm^2^) = (1.2 ± 0.3) × 10^3^· (*I*(D)/*I*(G))^−1^·*E*_*F*_^−0.54±0.04^/*E*_*L*_^4^, where *E*_*L*_ is the laser excitation energy in eV^40^. The Fermi energy, E_F_, can be estimated from a combined analysis of the 2D to G peak intensity ratio, *I*(2D)/*I*(G), the 2D to G area ratio, *A*(2D)/*A*(G), and the 2D peak position, Pos(2D)^34,77^. We fit Pos(2D) ~ 2693.8 ± 1.3 cm^−1^, *I*(2D)/*I*(G) ~ 1.4 ± 0.4, and *A*(2D)/*A*(G) ~ 2.8 ± 0.4. These correspond to *E*_*F*_ ~  − 0.4 ± 0.2 eV and a doping density ~ 1 × 10^13^ cm^−2^^34,77^. *I*(D)/*I*(G) ~ 0.8 ± 0.2 translates into ℓ_D_ ~ 9 ± 3 nm. From the histogram of *τ* in Fig. 2e the mean free path of carriers is ℓ_mfp_ = 11.3 nm, consistent with the average Raman defect distance. We note that the relations used are limited to Raman-active defects. Perfect zigzag edges^33,78^, charged impurities^34,38^, intercalants^79^, uniaxial and biaxial strain^22,80^ do not generate a D peak.

**Figure 4:**
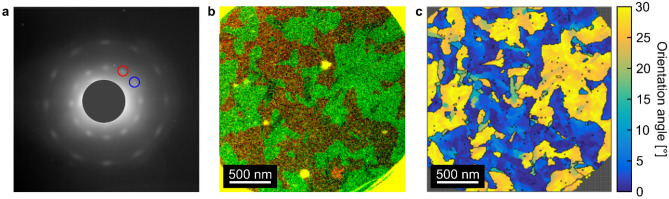
SED characterization of SLG transferred onto a TEM grid. (**a**) Spatially averaged diffraction pattern in which two sets of diffraction spots are present, indicating two main orientations. (**b**) Diffraction contrast image, showing the grain structure, formed by integrating the intensity within the red and green circles marked in (**a**) and superimposing the resulting images. (**c**) Orientation map plotting the absolute orientation angle of the SLG lattice at each probe position.

**Figure 5:**
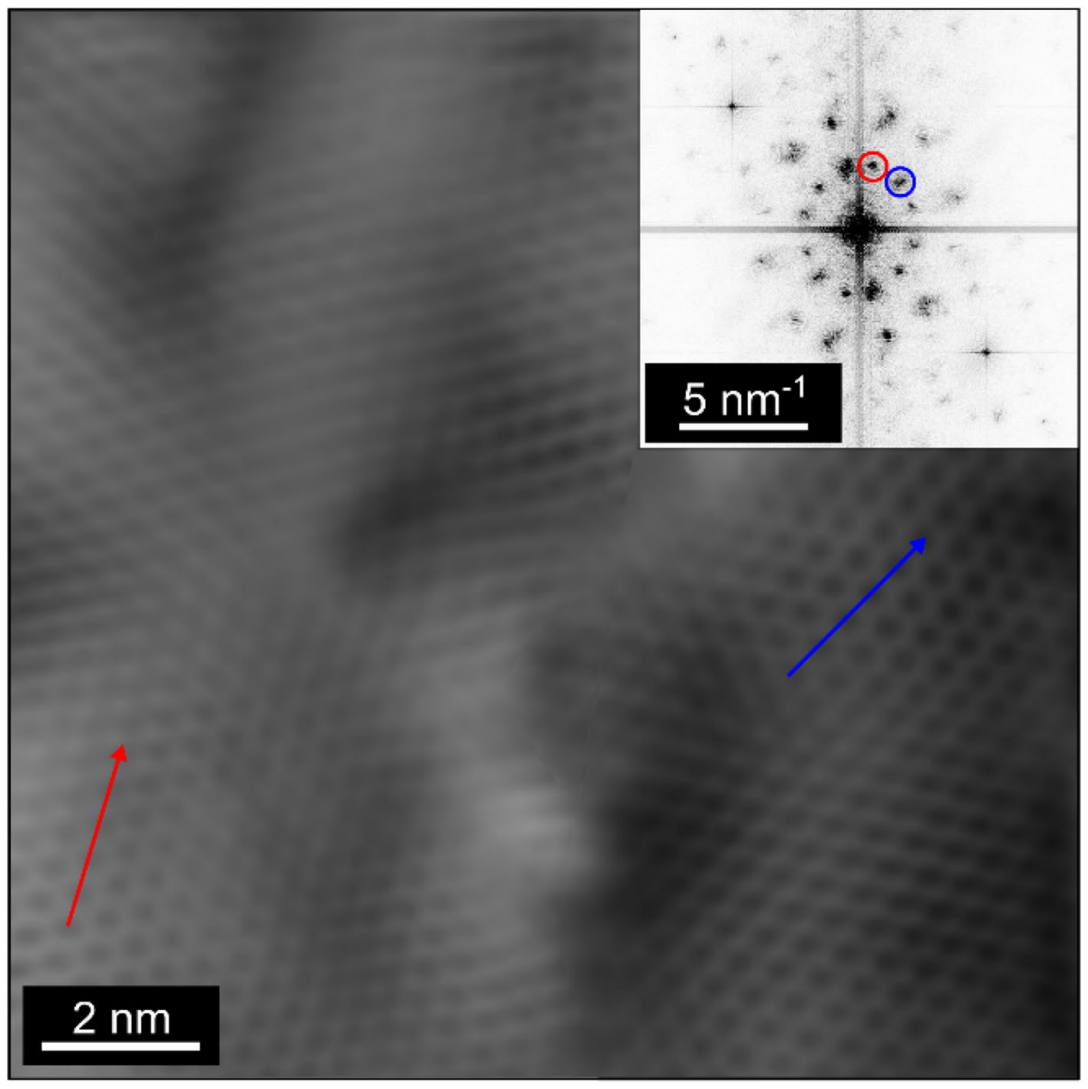
STM topography image filtered in frequency domain of SLG on n-doped Si showing a GB. The two SLG grains meet at a misorientation angle ∼29 ± 9°, seen in the two sets of diffraction peaks in the inset, and highlighted by the arrows showing the two distinct orientations.

SED and STM characterization are then carried out to inspect the GBs. Figure 4a plots the spatially averaged diffraction pattern calculated by summing all acquired patterns for a sample. Two sets of diffraction peaks with hexagonal geometry are observed, corresponding to two major orientations of the underlying SLG lattice within the scanned region. The diffraction peaks are however spread in the azimuthal direction indicating that there is variation around each of the major orientations with a magnitude ∼2°. The images formed by integrating the intensity of pixels within each of the circles marked in Fig. 4a yield the diffraction contrast images in Fig. 4b. Although there are only two major orientations within the scanned region, this reveals that there are many grains and GBs where they meet. The grains have a wide range of diameters ∼10–1000 nm, while the grain morphologies are irregular, and the GBs follow tortuous paths. This suggests that the prevalence of only two orientations is likely related to surface reconstructions of the substrate during growth. A grain orientation map (Fig. 4c) is derived from the diffraction contrast with GBs, defined when the pixel-to-pixel angular deviation is > 5°. Figure 4c shows that the misorientation angle between the two major orientations is ~ 30°, further supported by STM imaging (Fig. 5), where GBs meet at an angle ∼29 ± 9°. The SLG lattice is still visible after two transfer processes, although with nm-scale buckling of the SLG.

**Figure 6:**
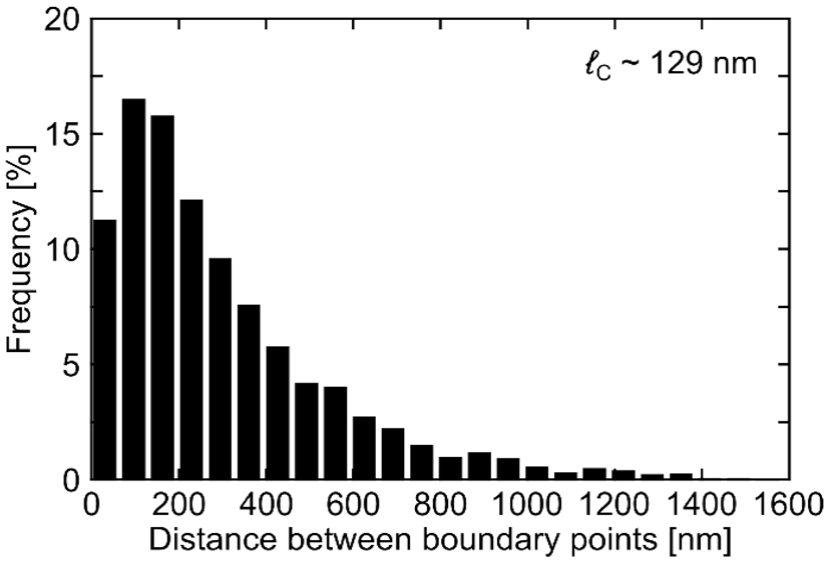
Histogram of GB distances sampled along 1000 randomly positioned test lines passing through the grain structure in Fig. 4b,c.

The distance between boundary points in Fig. 4c is shown in Fig. 6. The peak of the distance distribution from the Gaussian kernel density estimation method^82^ is ~ 129 nm, while in Fig. 2e, the distribution of scattering time above 60 fs (corresponding to 60 nm) is vanishing. ℓ_C_ derived from the GBs with large angle differences does not match ℓ_D_ from Raman spectroscopy nor ℓ_mfp_ from THz-TDS. This suggests that the large angle (~ 30°) GBs are not the main contributors to the strong reflective backscattering (*c* ~  − 0.9) in the DS model. In fact, the orientation angles appear to be distributed in two clusters, suggesting that SLG structured in larger grains with complex shapes and bimodal angles (0° orientation colored blue and 30° orientation colored yellow in Fig. 4c), where each of the two modes are subdivided into smaller grains with relative orientation angle variations of up to 5°, where the GB distance for the smaller grains will be closer to the distance between defects derived from Raman and the mean free path derived from THz-TDS. From the combination of Raman, SED, and THz-TDS it appears that even small angle orientation variations within SLG grains are detrimental for the electrical properties. While the SLG studied here is not immediately comparable to CVD SLG grown on conventional substrates (Cu, Ni and other transition metals), or prepared by exfoliation from bulk graphite, due to the spurious surface reconstructions, both experimental and theoretical studies find that that the GB resistance is strongly dependent on angle, with small misorientation angle GBs exhibiting the smallest GB resistance^83,84^. Ref.^85^ calculated GB resistance as a function of angle, finding it to be significantly smaller for small-angle misalignment, while Ref.^86^ suggested that non-straight GBs have substantially less misorientation angle dependence of the GB resistance at higher angles, while still decreasing rapidly ≤ 8°. The type of SLG studied here has an unusual bimodal GB distribution, Fig. 4c, which allows us to study the relative importance of small compared to large misorientation angles on electrical transport. In conjunction with the information on the grain structure provided by TEM and Raman spectroscopy, the THz-TDS measurements reveal that the microstructure of our CVD SLG leads to significant confinement-like effects, despite the < 5° misorientation angle observed with TEM. This needs to be considered to achieve consistent, reproducible, and cost-effective large scale SLG devices.

The correlation between carrier density (i.e. extracted locally by Raman spectroscopy or averaged over mm-sized regions by THz-TDS) and GB scattering is not considered here, but the latter is expected to depend strongly on the former^84^. It would be interesting to study whether the dominance of small misorientation angle GB scattering persists at lower or higher carrier densities, e.g. with a global, THz-transparent back gate^32^.

## Conclusions

The electrical continuity of polycrystalline graphene can be studied by THz-TDS by fitting the conductivity with the Drude–Smith model. In this work, we mapped the Drude–Smith backscattering parameter across a graphene-coated Si wafer. Comparison to Raman and SED measurements indicates that carrier scattering in SLG may be strongly influenced by small angle orientation variations < 5° within graphene grains, leading to non-isotropic scattering with characteristic length scales below the average distance between large-angle grain boundaries. THz-TDS allowed us to evaluate the carrier mean free paths in the nanoscale range, as well as the nanoscale structure of SLG, validated by two independent confirmations of the characteristic length scale in the nanoscale range, consistent with the non-zero backscattering parameter c in the Drude–Smith like THz-response. These results (1) link characteristic transport lengths in small-grain graphene obtained with THz-TDS with two other methods (Raman and TEM); (2) correlate carrier mobility with the backscattering parameter; (3) show that even small-angle misalignment of graphene grains can lead to strong carrier confinement. Our work highlights that grain boundary control is important to optimize performance in graphene devices, circuits, and related technologies.

## Data Availability

The datasets generated and used during the current study are available from the corresponding author on reasonable request.
